# SNPs and real-time quantitative PCR method for constitutional allelic copy number determination, the *VPREB1 *marker case

**DOI:** 10.1186/1471-2350-12-61

**Published:** 2011-05-05

**Authors:** Marcello Frigerio, Elena Passeri, Tiziana de Filippis, Daniela Rusconi, Rea Valaperta, Mario Carminati, Anita Donnangelo, Elena Costa, Luca Persani, Palma Finelli, Sabrina Corbetta

**Affiliations:** 1 Research Laboratories - Molecular Biology, IRCCS Policlinico San Donato, San Donato Milanese (MI), Italy; 2 Endocrinology and Diabetology Unit, IRCCS Policlinico San Donato, San Donato Milanese (MI), Italy; 3 Department of Medical-Surgical Sciences, Università di Milano, San Donato Milanese (MI), Italy; 4 Laboratorio di Ricerche Endocrino-Metaboliche, IRCCS Istituto Auxologico Italiano, Milan, Italy; 5 Department of Medical Sciences, Università di Milano, Milan, Italy; 6 Laboratory of Medical Cytogenetics and Molecular Genetics, IRCCS Istituto Auxologico Italiano, Cusano Milanino (MI), Italy; 7 Department of Biology and Genetics for Medical Sciences, Università di Milano, Milan, Italy; 8 Pediatric Cardiology and Adult with Congenital Heart Disease Department, IRCCS Policlinico San Donato, San Donato Milanese (MI), Italy

**Keywords:** qRT-PCR, allelic copy number, 22q11.2 microdeletion, DiGeorge Syndrome

## Abstract

**Background:**

22q11.2 microdeletion is responsible for the DiGeorge Syndrome, characterized by heart defects, psychiatric disorders, endocrine and immune alterations and a 1 in 4000 live birth prevalence. Real-time quantitative PCR (qPCR) approaches for allelic copy number determination have recently been investigated in 22q11.2 microdeletions detection. The qPCR method was performed for 22q11.2 microdeletions detection as a first-level screening approach in a genetically unknown series of patients with congenital heart defects. A technical issue related to the *VPREB1 *qPCR marker was pointed out.

**Methods:**

A set of 100 unrelated Italian patients with congenital heart defects were tested for 22q11.2 microdeletions by a qPCR method using six different markers. Fluorescence In Situ Hybridization technique (FISH) was used for confirmation.

**Results:**

qPCR identified six patients harbouring the 22q11.2 microdeletion, confirmed by FISH. The *VPREB1 *gene marker presented with a pattern consistent with hemideletion in one 3 Mb deleted patient, suggestive for a long distal deletion, and in additional five non-deleted patients. The long distal 22q11.2 deletion was not confirmed by Comparative Genomic Hybridization. Indeed, the *VPREB1 *gene marker generated false positive results in association with the rs1320 G/A SNP, a polymorphism localized within the *VPREB1 *marker reverse primer sequence. Patients heterozygous for rs1320 SNP, showed a qPCR profile consistent with the presence of a hemideletion.

**Conclusions:**

Though the qPCR technique showed advantages as a screening approach in terms of cost and time, the *VPREB1 *marker case revealed that single nucleotide polymorphisms can interfere with qPCR data generating erroneous allelic copy number interpretations.

## Background

Real-time quantitative PCR (qPCR) performed with standard curves has been proposed as a routine, reliable and highly sensitive assay for gene expression analysis [1]. qPCR applications are as well becoming a reference method, alternative to Southern blot and Fluorescence In Situ Hybridization (FISH), for the measurement of gene copy number in human tumours with allelic imbalances [2]. Similar applications have been finally adopted for constitutional allelic copy number determination with several examples in the detection of 22q11.2 microdeletions responsible for the homonymous syndrome, also known as DiGeorge syndrome (DGS; OMIM 188400) [3-7]. DGS encompasses a wide spectrum of clinical features with variable expression including dysmorphic facies, palate abnormalities, congenital conotruncal cardiac defects, endocrine dysfunctions, T-cell mediated immune deficiency and psychiatric disorders [8]. This autosomal dominant disorder constitutes a breeding ground for the development of a real-time qPCR based technique because of its frequency (1:4000 live births) and its allelic heterogeneity. Indeed, the DGS clinical phenotype may result from multiple types of microalterations at the same locus including the most frequent 3 Mb deletion and the smaller nested 1.5 Mb deletion, occurring respectively in approximately 90% and 7% of patients with a 22q11.2 anomaly, and other atypical and rare microdeletions and microduplications affecting the critical 3 Mb region and its proximal and distal flanking regions [9].

The specific qPCR analysis for the 22q11.2 region allelic copy number determination performed with SYBR Green chemistry has been described by Weksberg [6]. It based on the comparison among the amplifications of 10 different markers localized within the deletion critical 22q11.2 sequence and its proximal regions affected with rare recently described distal deletions [9]. As the SYBR Green molecule is a fluorescent non-specific intercalant agent, the primers design represents the essential point for the method specificity. The qPCR primers were designed within regions of unique sequence avoiding the complex repetitive regions, possibly within gene exons sequences [6,7].

The qPCR method was previously proposed as a sensitive technique for precise deletion breakpoints definition with theoretical useful applications in genotype-phenotype correlation studies [6,7]. Indeed, the qPCR approach could be applied to patients with an evident syndromic phenotype who are not diagnosed with clinical standard cytogenetic methods due to atypical alterations. Considering its intrinsic technical advantages, we tested the Weksberg qPCR method for 22q11.2 microdeletion detection as a first-level screening technique exploitable for wide population studies and useful as screening approach to the high-resolution cytogenetic tools such as Comparative Genomic Hybridization (CGH) arrays.

In the present study the Weksberg's qPCR approach for 22q11.2 microdeletion detection was performed in a wide genetically unknown series characterized by a phenotypic trait (congenital heart defects) included in DGS clinical features. A technical issue related to the *VPREB1 *qPCR marker was pointed out.

## Results

### Real-time quantitative PCR results

Eighty-eight patients presented a qPCR markers profile similar to that observed in del22q11.2 negative control samples indicating no loss in allelic copy number.

Six patients, five unrelated and a homozygotic twin, harboured a microdeletion as they showed a qPCR markers combination suggestive of a long distal deletion. In these patients *D22S181 *and *VPREB1 *amplifications were consistent with the presence of two allelic copies, whereas the amplification profile of *PRODH*, *TUPLE1*, *COMT *and *D22S936 *indicated a loss of one allelic copy. Presence of microdeletions was confirmed in all the six patients with a standard FISH analysis.

An unexpected result from the patients screening was the finding in five patients of qPCR marker profiles characterized by the only *VPREB1 *hemideletion, suggestive for the presence of atypical distal deletions. One further patient (patient no.4 in Table 1; index case) showed a qPCR set with hemideletion in the *PRODH*, *TUPLE1*, *COMT*, *D22S936 *and *VPREB1 *markers that could be consistent with a long atypical deletion.

**Table 1:** Overview of the qPCR markers results

Patients	**No**.	22q11.2 region qPCR markers results (ΔKC_t_)					
		***D22S181***	***PRODH***	***TUPLE1***	***COMT***	***D22S936***	***VPREB1***

**Not deleted**	88	0.007 ± 0.123	-0.009 ± 0.006	-0.018 ± 0.076	0.039 ± 0.009	-0.002 ± 0.062	0.082 ± 0.008
**22q11.2** **hemideletion**	6	-0.004 ± 0.140	**-0.982 ± 0.039**	**-1.076 ± 0.014**	**-0.958 ± 0.154**	**-0.887 ± 0.047**	0.080 ± 0.024
Patient no.4	1	0.106 ± 0.014	**-0.992 ± 0.025**	**-1.037 ± 0.069**	**-0.994 ± 0.104**	**-0.918 ± 0.001**	**-1.050 ± 0.186**
**VPREB1 hemideletion**	5	0.067 ± 0.087	0.005 ± 0.098	0.038 ± 0.095	0.005 ± 0.115	0.020 ± 0.110	**-1.093 ± 0.042**

Data are expressed as the mean ΔKC_t _± standard deviation calculated on all the subjects of each patients set. ΔKC_t _consistent with a loss of one allelic copy are indicated in bold.

*VPREB1 *marker standard curves and melting curves analysis performed in all subjects presenting the *VPREB1 *marker hemideletion was consistent with the 10 del22q11.2 negative controls profile thus indicating no difference in primer efficiency and amplicon specificity.

### Fluorescence *in situ *hybridization and Array CGH on index case DNA

Standard FISH analysis confirmed the presence of a microdeletion in the 22q11 region in the index case. The patient's DNA was further investigated by CGH assay to test the qPCR-based deletion extension hypothesis.

**Figure 1:**
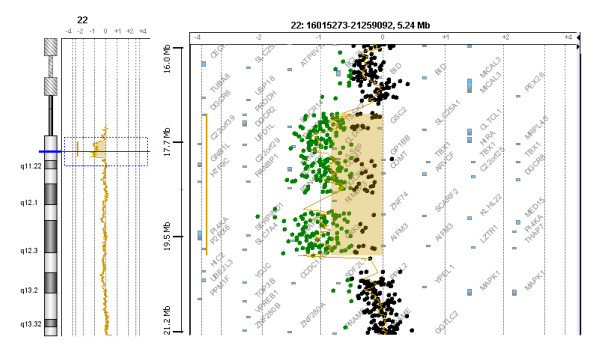
**Array CGH profile of index case DNA**. A) Whole chromosome 22 array profile is shown. Scattered plot analysis reveals a deletion in 22q22.12 (horizontal shift to left of 0). B) Zoomed-in gene view of panel A which focuses on a 5.2 Mb window within 22q22.12 containing the deletion. Each point represents a single probe. Log_2 _(ratio) was plotted for all oligonucleotide probes based on their chromosome positions. Aberration calls identified by ADM-2 algorithm (coloured shaded areas) are shown.

**Figure 2:**
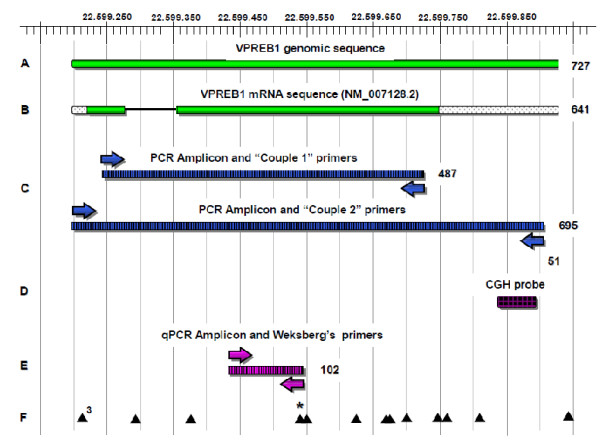
**Schematics of the *VPREB1 *gene 22q11.2 region**. A) Genomic sequence of *VPREB1 *gene. B) *VPREB1 *mRNA sequence (NM_007128.2), intron sequence was represented with a black line, dotted ends indicated untranslated regions. C) PCR products resulting from Set 1 and Set 2 primers amplification. D) CGH-array *VPREB1 *probe. E) *VPREB1 *Weksberg's primers and relative qPCR product. F) Locations of SNPs, the rs1320 polymorphism is highlighted with a (*). For A-E, the number on the right indicates the base pairs present in each relative segment.

Array CGH revealed a heterozygous deletion on chromosome 22q11.21 spanning at least 2.56 Mb (chr22: 17,274,835-19,835,417, hg18; chr22: 18,894,835-21,505,417, hg19 UCSC) (Figure 1). The centromeric breakpoint maps between probe A_16_P41477672 and probe A_16_P41477688 (Chr22: 17,270,271-17,274,894 bp, UCSC, hg18; chr22: 18,890,271-18,894,894, hg19 UCSC) in LCR22-2, whereas the distal breakpoint maps between probe A_16_P41484416 and probe A_16_P03603092 (Chr22: 19,835,358-19,891,514 bp, UCSC, hg18; chr22: 21,505,358-21,561,514, hg19 UCSC) in LCR22-4. CGH assay confirmed the presence of the classical deletion. Nor the critical region distal probes nor the specific *VPREB1 *probe (Figure 2, line D) of the CGH-array showed the presence of a hemideletion in *VPREB1 *gene or in its proximal region.

### *VPREB1 *genotype results

*VPREB1 *gene sequence analysis performed with Set 1 primers (Table 1 and Figure 2, line C) on the six subjects with hemideletion in *VPREB1 *marker showed the presence of heterozygosity for the rs1320 A/G SNP, a rare (minor allele frequency in the European Population = 0.062) missense single nucleotide polymorphism [10] localized within the complementary region of the qPCR *VPREB1 *marker reverse primer. This finding was consistent with the interpretation of the qPCR *VPREB1 *marker hemideletions as false positive results.

The subsequent sequencing with Set 2 primers (Table 2 and Figure 2, line C) of the remaining 94 subjects with a qPCR *VPREB1 *marker were consistent with the presence of two allelic copies demonstrating that all patients were homozygous carriers of the rs1320 ancestral G allele.

**Table 2:** Sequences and locations of *VPREB1 *PCR amplification primers

Primer Sets	Primer Sequences	Amplicon Size	Genomic Location of Amplicon
**Set 1**	AGAGCTCTGCATGTCTGCAC	695	22599205-22599900 (Chr 22)
	TTCCCTAATGCAGTCTCCAA		
**Set 2**	TCCTGCTCATGCTGTTTGTC	487	22599242-22599729 (Chr 22)
	CTGCAGTGGGTTCCATTTCT		

No immunological disorder was found to be associated with the rs1320 A allele.

## Discussion

The real-time quantitative PCR approach could represent a robust, fast and accurate assay for the detection of copy number alterations in genomic DNA [2-7]. Two peculiar advantages of the qPCR method have been focused: the detection of atypical microdeletions undiagnosed by diagnostic standard FISH approach and the accurate mapping of deletion breakpoints. Previous studies on the real-time quantitative application to 22q11.2 microdeletions detection [6,7] were performed in small series with a previous cytogenetic analysis positive for 22q11.2 microdeletions, as the main purpose was the refinement of the deletion breakpoints maps with the increasing of the number of markers in the 22q11.2 deleted region.

In the present study, we performed the qPCR method in a large paediatric genetically unknown patient cohort screening. We selected a reduced number of 22q11.2 region markers to provide a rapid patient genotype. The six markers effectively identified and differentiated the typical 22q11.2 alterations (the 3 Mb and the 1.5 Mb microdeletions) and the rare atypical microdeletions. Our results confirmed the efficiency of the method in an unselected series of young patients with congenital heart defects, identifying six patients harbouring the classic 3 Mb microdeletion fully concordant with the FISH analysis results. Interestingly, this approach provided DGS diagnosis in patients with atypical mild clinical presentations, extending the accuracy of the diagnosis and allowing genetic counselling.

At variance with the data presented by Weksberg et al. [6,7], the *VPREB1 *qPCR marker analysis produced results suggestive for the presence of distal deletions at an unexpected high frequency. Nonetheless, the long extension of the deletion was not confirmed by the CGH analysis. Investigating the molecular basis of these discrepancies, we considered that the *VPREB1 *gene sequence is characterized by a high frequency of SNPs and that the Weksberg's qPCR *VPREB1 *marker reverse primer sequence was specifically designed for the exon 2 *VPREB1 *sequence containing the rs1320 SNP G ancestral allele.

Our data suggested that the presence in heterozygosity of the rs1320 A allele constitutes a critical mismatch in the reverse primer sequence leading to an inefficient primer annealing and a consequent misamplification. Indeed, under these assumptions, patients with an heterozygous profile for rs1320, that are carriers of a single fully complementary allelic copy for qPCR *VPREB1 *marker amplification, should produce, as detected in our experience, a ΔKC_t _value range similar to that owned by patients with a constitutional single allelic copy due to microdeletion. This hypothesis resembles similar experiences previously described [11,12].

## Conclusions

The *VPREB1 *qPCR marker case here described underscored the effect of a single nucleotide substitution in the primer complementary sequence that can lead to an invalid qPCR amplification resulting in a defective allelic copy number interpretation. From this observation we take advantage to stress the relevance of the primer design for any kind of real-time PCR using standard curves analysis. Primer sequences should be designed avoiding both the repeated sequence regions and the high polymorphic loci.

Finally, data here presented highlighted how this qPCR method could be a rapid, cost-effective and easy to reproduce assay for 22q11.2 region genotyping when applied with a confined number of markers on a high-throughput system. These advantages are desirable for an efficient first-level screening approach. We feel that the qPCR approach could represent a valid alternative to the more classical and expensive cytogenetic analysis, and therefore a helpful clinical tool for the 22q11 screening in patients with a non-classic phenotype.

## Methods

### Subjects

qPCR method was adopted for the screening of a retrospective cohort of 100 consecutive unrelated Italian paediatric patients [48 females, 52 males; 6.51 (4.48-9.06) years (median age, IQ range)] referred to the Paediatric Cardiac Surgery Unit of the IRCCS Policlinico San Donato between 2007 and 2009 for the management of congenital heart defects. The congenital heart defects were: atrial septal defects (28%), ventricular septal defects (20%), patent ductus arteriosus (15%), aortic coartations (14%), Tetralogy of Fallot (12%), miscellaneous uncommon lesions (11%). Patients affected with known genetic syndrome were excluded. The genetic study was adherent to the principles of Helsinki Declaration. Parents of all participants signed an informed consent for genetic testing.

### Real-time quantitative PCR (qPCR)

**Figure 3:**
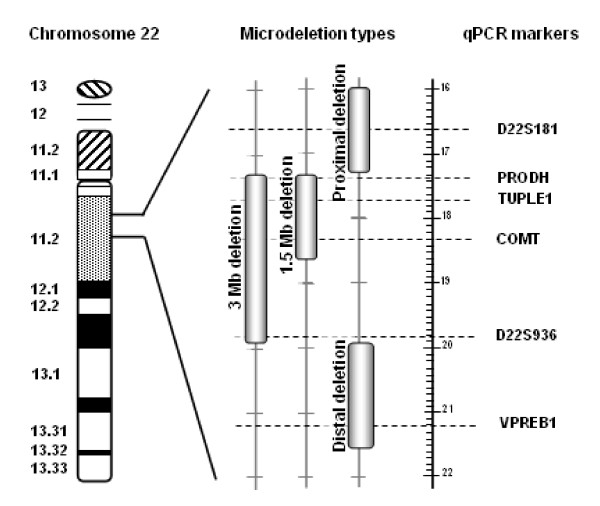
**Representation of Chromosome 22 with enlargement of the 22q11.2 region and qPCR markers locations**. qPCR markers localization was shown with respect to the two most classical 3 Mb and 1.5 Mb microdeletions and two different examples of atypical deletions. *PRODH*, *TUPLE1 *and *COMT *markers recognized 3 Mb and 1,5 Mb microdeletions; *D22S936 *marker allowed to distinguish between 3 Mb or 1.5 Mb microdeletions; *D22S181 *and *VPREB1*, both localized outside the critical deletion region, allowed the detection of respectively proximal and distal atypical microalterations.

Genomic DNA was extracted using standard procedures from EDTA-blood samples collected from patients at the time of admission after an overnight fasting. Real-time quantitative PCR analysis was performed with the *D22S181*, *PRODH*, *TUPLE1*, *COMT*, *D22S936 *and *VPREB1 *markers to obtain an immediate overview of the patient genotype (Figure 3). Primer sequences were derived from Weksberg studies [6,7]. Though the marker sets used in the present study were reduced from those reported by Weksberg [6,7], the efficient discrimination of the two most frequent alterations, the 3 Mb and the 1.5 Mb hemizygous deletions, and the detection of atypical deletions were ensured. *G6PDH *and *HEM3 *markers were also amplified for the subsequent data normalization.

Real-time qPCR assays were performed in a 25 μl reaction volume containing 12.5 μl 2× SYBR Green I PCR Master Mix (Applied Biosystems, Foster City, CA, USA), 10 ng genomic DNA, forward and reverse primers at final concentration of 800 nM for the specific 22q11.2 region markers and 400 nM for *G6PDH *and *HEM3 *markers. Reactions were performed in 96-well MicroAmp Optical Reaction Plates (Applied Biosystems) using the ABI Prism 7900 high-throughput sequence detection systems (Applied Biosystems) and data were processed by the associated SDS software version 2.3 (Applied Biosystems). Standard curves for all the primers set used were generated with series of log dilution of 4 del22q11.2 negative samples genomic DNA. Slopes derived from standard curves were used for data normalization following Weksberg's instructions [6]. Reaction specificity was confirmed with melting curves analysis and agarose gel electrophoresis experiments. Each qPCR assay included at least 3 del22q11.2 negative control samples; all reactions were performed in triplicate, with replicates being performed on different days. Allelic copy number of the 22q11.2 region markers where finally determined with the comparison of normalized data of control samples and patients. Final ΔKC_t _values were considered as follows: the interval -0.75 to -1.25 indicated loss of one copy (presence of hemizygous microdeletion), whereas a ratio of 0.00 ± 0.25 corresponded to two allelic copies (no microdeletion). Data normalization and allelic copy number equations were derived from Weksberg's studies [6,7].

All qPCR markers presented at the standard curves analysis an amplification efficiency similar to that reported by Weksberg [6], however each standard curve slope value was found in the 3.32 ± 0.25 range allowing the successive amplification comparison and data elaboration. Melting curve analysis and agarose gel-electrophoresis assay confirmed the amplicons specificity showing respectively a single peak on the dissociation curve plot and a single band of the expected size on the electrophoretic run. qPCR results for this study series are summarized in Table 1.

### Fluorescence *in situ *hybridization analysis

The FISH technique was performed on peripheral blood lymphocytes according to the manufacturer's instruction. A commercial probe results in the simultaneous labelling of the 22q11.2 chromosome region that includes *TUPLE1 *gene also known as HIRA gene (red labelling) and the terminal region of chromosome 22 as control (green labelling) was used. Twenty patient's metaphases were analyzed under a fluorescence microscope (Leica Microsystem, Wetzlar, Germany).

### Array CGH analysis

For CGH, genomic DNA was extracted from proband's blood using the Genelute Blood Genomic DNA Kit (Sigma-Aldrich Corp. St. Louis, MO, USA) according to the manufacture's instructions. Pooled DNA from the peripheral blood of 10 healthy donors (Promega, Madison, WI), sex-matched to the sample was used as a reference for the DNA extracted from peripheral blood. Genomic DNA (1.5 μg) was hybridized to the 244 KB microarray (Agilent Technologies, Palo Alto, CA, USA), consisting of ~236,000 60-mer oligonucleotide probes covering the entire genome at the average spatial resolution of ~30 Kb, and processed according to the manufacturer's instructions.

The dye emission capture was performed by a dual-laser Agilent Scanner. Images were extracted using Agilent Feature Extraction software 9.1 and analysed by DNA Analytics 4.0 software. A log ratio plot between test and reference genomic DNA was assigned such that aberrations in test DNA copy number at a particular locus are observed as the deviation of the ratio plots from a modal value of 0; aberration calls was identified by the ADM-2 algorithm.

### *VPREB1 *Sequencing

*VPREB1 *amplification was performed on all qPCR analysed samples with the same genomic DNA template used in qPCR experiments. Two different primers sets were designed using Primer3 online software [13]. Set 1 amplicon performed the sequencing of the entire *VPREB1 *gene, while Set 2 amplicon was centred within the qPCR marker reverse primer sequence (Figure 2). Primer sequences are shown in Table 2.

PCR was performed in a 25 μl reaction volume containing 200 ng genomic DNA, 2.5 U Platinum Taq DNA Polymerase (Invitrogen, Madison, WI, USA), 25 μmol of each primer, 100 μmol/L of each dNTP, 1× PCR buffer (Invitrogen) and 1.5 mM MgCl_2 _with the use of a MyCycler thermal cycler (Biorad, Hercules, CA, USA). Cycling variables were as follows: 94°C for 5 min (first denaturing step); 30 cycles of 94°C for 1 min, 56.3°C for 1 min and 72°C for 2 min; and 72°C for 10 min (last extension step). We did bidirectional sequencing of the purified PCR products (GE Healthcare, Buckinghamshire, UK) by a BigDye terminator sequencing kit (Applied Biosystems) and a 3100 genetic analyser (Applied Biosystems). Sequences were analysed by the ABI Prism Sequencing Analysis Software (version 3.7, Applied Biosystems).

## Competing interests

The authors declare that they have no competing interests.

## Authors' contributions

MF conceived the study, participated in its design and coordination, carried out the real-time qPCR studies and drafted the manuscript. TF and LP participated in the VPREB1 gene sequencing. DR and PF carried out the cytogenetic study. RV and AD participated in molecular genetics studies and, together with EC, participated in the coordination of the study and helped to draft the manuscript. EP contributed with pertinent clinical information and, together with SC, contributed to overall study design. All authors read and approved the final manuscript.

## Pre-publication history

The pre-publication history for this paper can be accessed here:

http://www.biomedcentral.com/1471-2350/12/61/prepub
